# Oxidative stability and lipid oxidation flavoring volatiles in antioxidants treated chicken meat patties during storage

**DOI:** 10.1186/s12944-017-0426-5

**Published:** 2017-02-01

**Authors:** Muhammad Sohaib, Faqir Muhammad Anjum, Muhammad Sajid Arshad, Muhammad Imran, Ali Imran, Shahzad Hussain

**Affiliations:** 1 Department of Food Science and Human Nutrition, University of Veterinary and Animal Sciences, Lahore, 54000 Pakistan; 2 Institute of Home and Food Sciences, Faculty of Science and Technology, Government College University, Faisalabad, 38040 Pakistan; 3 Department of Food Science, Nutrition and Home Economics, Government College University, Faisalabad, Pakistan; 4 Department of Food Science and Nutrition, College of Food and Agricultural Sciences, King Saud University, Riyadh, Saudi Arabia

**Keywords:** Antioxidants, Quercetin dihydrate, Volatiles, Hexanal, TBARS, Total carbonyls, Storability

## Abstract

**Background:**

Chicken meat contains higher percentage of polyunsaturated fatty acids that are susceptible to oxidative deterioration ultimately leading towards lower consumer acceptability for chicken meat products. Accordingly, meat processing industries are looking for combinations of natural antioxidants to enhance the oxidative stability and consumer acceptability of meat based products. The present study aimed to investigate the influence of directly added quercetin dihydrate in combination with α-tocopherol on oxidative stability, color characteristics, total carbonyls and flavor volatile compounds in chicken meat patties.

**Methods:**

Considering the preliminary studies, 3 levels of quercetin dihdrate @ 25, 50 and 100 mg/kg meat in combination with α-tocopherol at the rate 100 and 200 mg/kg meat were added to develop chicken meat patties and were stored at refrigeration temperature for 7 days. The oxidative stability of the antioxidant treated patties was determined by measuring malonaldehydes using TBARS and total carbonyls assay. The color (Lightness, redness and yellowness) of the patties was determined by using Konica Minolta Color Meter. Moreover, the volatile compounds were measured through gas chromatography at various storage intervals.

**Results:**

The results elucidated that quercetin dehydrate inclusion at the rate of 50 mg/kg meat as well as particularly 100 mg/kg meat decreased the oxidation by reducing generation of malonaldehydes and total carbonyls in treated patties. Highest value for TBARS at initiation of storage was reported in (T_0_) as 1.93 ± 0.02 whereas lowest were reported in T_6_ and T_5_ as 0.37 ± 0.01 and 0.38 ± 0.03 that were increased to 3.47 ± 0.14, 0.90 ± 0.05 and 0.94 ± 0.34 at the completion of storage. Moreover, the lowest carbonyls also reported in T6 and the values at various storage intervals (1st, 3rd and 7th) were as 0.59 ± 0.025, 0.77 ± 0.015 and 1.02 ± 0.031, respectively. The antioxidants inclusion also inhibited volatile flavoring compounds particularly aldehydes like hexanal and pentanal in a dose dependent manner (*p* ≤ 0.05). Lowest hexanal values reported in T_6_ as 2488 ± 103 followed by T_4_ (3701 ± 111) at the start of the trial whereas highest in T_0_ (control) as 54,768 ± 431 that were increased to 9569 ± 607, 112,550 ± 897 and 359,826 ± 1285, correspondingly. The hexanal, as a critical indicator for the determination of volatiles in meat based products, was decreased with the addition of antioxidants and its highest values were reported in control group.

**Conclusions:**

Quercetin dihydrate addition along with alpha tocopherol is a pragmatic choice to improve oxidative storability and volatile flavor compounds in cooked meat patties. The data obtained will help meat processor to better develop antioxidant enriched formulations to augment oxidative stability and quality of processed meat products.

## Background

Lipid oxidation represents one of the most important causes of deterioration in meat and meat products and it affects unsaturated fatty acids particularly polyunsaturated fatty acids (PUFA) in membrane phospholipids as well as cholesterol, mainly low density lipoprotein (LDL) cholesterol. The final end-products of this process can damage the aroma, color, flavor as well as sensorial attributes of meat and allied products; hence reduce the nutritive value [1]. Besides nutritional deterioration, lipid oxidation generates cytotoxic and genotoxic compounds which are deleterious for humans health [2]. The oxidative damage to meat based products results in problems like tissues damaging, putrification, loss of nutrients, enhanced free radical generation and malonaldehydes production that reduce the antioxidant capacity of products [3]. Lipid stability of meat mainly depends on the balance of antioxidants, oxidation substrate, cholesterol content as well as heme pigment.

The quality defects caused by the oxidative process in meat and meat products can be controlled by using antioxidant based formulations and their incorporation disrupts the oxidation chain reaction that further progress with passage of time ultimately reduces quality of meat based products [4]. The quercetin is one of the flavonol and a potent antioxidant having ability to reduce lipid peroxidation in meat based products by limiting the oxidation chain reaction. The antioxidant activity of quercetin is attributed to its ability to scavenge free radicals, donate hydrogen atoms or electrons or chelate metal cations. Additionally, various in vitro and in vivo studies conducted on experimental animals revealed quercetin antioxidant and anti-inflammatory prospectives [5]. The oxidative stability of protein and lipids in meat and meat based products can be improved by the addition of α-tocopherol due to its capacity to enhance the activity of cellular antioxidant enzymes. Moreover, addition of this antioxidants also improve color characteristics and overall quality attributes of chicken meat products [6]. It is also an essential micronutrient for maintaining the health and wellbeing of living organism due to antioxidant properties. Alpha tocopherol is the active form of vitamin E that is used in commercial formulations [7]. The addition of antioxidants rich formulations in various fresh and cooked meat products have potential to reduce oxidation problems by hindering the formation of free radicals. These additions beyond providing the protection against oxidative damage to meat products, also improve the safety and overall quality of processed meat products. Currently, meat processing industries are looking for natural antioxidant based formulations to enhance storability and volatile flavor compounds in cooked meat products. There is a dire need to develop formulations to be directly incorporated in meat products to retard lipid and protein oxidation as well as off flavor volatile compounds production. Considering these facts, present study was carried out to evaluate how much quercetin dihydrate alone as well as in combination with α-tocopherol can be added to increase the oxidative stability as well as formation of off-flavor volatiles in chicken meat patties.

## Methods

### Preparation of raw material and chemicals

To carry out current study, chicken meat was purchased from a local superstore. Moreover, Quercetin dihydrate was procured from Alfa Aesar (Johnson Matthey Company, Massachusetts, USA). All other chemicals and reagents required were obtained from Sigma Aldrich (Japan) and Merck (Germany).

### Processing and application of antioxidants treatments in patties

The meat was ground twice through a 10-mm and a 3-mm plate (Kitchen Aid, Inc., St. Joseph, MI, USA) before the application of antioxidants. Based on the preliminary studies, following treatments were made; T_0_ = Control without antioxidants; T_1_ = 25 mg quercetin dihdrate + 100 mg α-tocopherol/kg meat; T_2_ = 25 mg quercetin dihdrate + 200 mg α-tocopherol/kg meat; T_3_ = 50 mg quercetin dihdrate + 100 mg α-tocopherol/kg meat; T_4_ = 50 mg quercetin dihdrate + 200 mg α-tocopherol/kg meat; T_5_ = 100 mg quercetin dihdrate + 100 mg α-tocopherol/kg meat; T_6_ = 100 mg quercetin dihdrate + 200 mg α-tocopherol/kg meat. The quercetin dihydrate was dissolved in alkaline water for complete solublization. Moreover, α-tocopherol is prepared by dissolving in corn oil before initiation of experiment. The aforementioned treatments were incorporated to the ground meat followed by mixing for 2 min using bowl mixer (Model KSM 90, St. Joseph, MI, USA). Afterwards, patties (100 ± 3 g) were prepared, vacuum packaged in oxygen impermeable bags (O_2_ permeability, 9.3 mL O_2_/ m^2^/ 24 h at 0 °C, Koch, Kansas City, MO, USA) and cooked at 95 °C water bath (Fisher Scientific Inc., PA, USA) until the internal temperature reached to 75 °C. The cooked patties were cooled and repacked in new oxygen permeable bags (polyethylene, 4 × 6.2 mil, Association Bags Co., Milwaukee, WI, USA) and stored at 4 °C. The analyses of patties were carried out at 1st, 3rd and 7th day of storage.

### Analysis of patties

#### 2-Thiobarbituric acid reactive substances (TBARS) analysis

The oxidative stability of breast meat patties was estimated by using 2-thiobarbituric acid reactive substances (TBARS) according to the guidelines of [8]. In this context, 5 g of ground broiler meat samples were weighed in a 50 mL test tube and homogenized with 50 μL of butylated hydroxytoluene (7.2%) and 15 mL of deionized distilled water using a homogenizer for 15 s. One mL of meat homogenate was transferred to a disposable test tube (13 × 100 mm) and 2 mL of TBA/trichloroacetic acid (TCA; 15 mM TBA/15% TCA) solution was added. The mixture was vortex and incubated in a boiling water bath for 15 min to develop color. Afterwards, samples were cooled in ice water for 10 min, vortex again and centrifuged for 15 min at 2000 × *g* at 4 °C. The absorbance of the resulting supernatant solution was determined at 531 nm against a blank containing 1 mL of deionized distilled water and 2 mL of TBA/TCA solution. The amounts of TBARS were expressed as milligrams of malondialdehyde (MDA)/kg meat.

### Color measurement

The surface color of patties enriched with quercetin and α-tocopherol was measured using Hunter-Lab Mini Scan XE colorimeter (Hunter Laboratory Inc., Reston, VA) with D_65_ illuminant and 10° standard observer. Three readings for every sample were obtained and averaged for Commission Internationale d’Eclairage L* (lightness), a* (redness) and b* (yellowness) of patties.

### Volatile compounds

The volatile compounds of cooked meat patties were measured through Solatek 72 Multimatrix-Vial Auto-sampler/Sample Concentrator 3100 (Tekmar-Dohrmann, Cincinnati, OH, USA) connected to GC/MS (Model 6890/5973; Hewlett-Packard Co., Wilmington, DE, USA) according to the method of [9]. Purposely, 2 g meat sample was placed in 40 mL sample vial, flushed with helium gas (40 psi) for 3 s and capped airtight with a Teflon*fluorocarbon resin/silicone septum (I-Chem Co., New Castle, DE, USA). The samples of different treatment were randomly organized on the refrigerated (4 °C) holding tray to minimize the variation of the oxidative changes in samples. The meat samples were purged with helium (40 mL/min) for 14 min at 20 °C. The volatile compounds were trapped using Tenax/charcoal/silica column (Tekmar-Dohrmann) and desorbed for 2 min at 225 °C, maintained in a cryofocusing module (−70 °C) and then thermally desorbed into a capillary column for 2 min at 225 °C. The HP-624 column (7.5 m, 0.25 mm i.d., 1.4 mm), HP-1 column (52.5 m, 0.25 mm i.d., 0.25 μm) and HP-Wax column (7.5 m, 0.250 mm i.d., 0.25 μm) were connected through zero dead volume column connectors (J &W Scientific, Folsom, CA, USA). Initially, the oven temperature was 25 °C for 5 min and increased to 85 °C at the rate of 40 °C per min leading to 165 °C at the rate of 20 °C per min and finally 230 °C at 5 °C per min. held at this temperature for 2.5 min. The constant column pressure 22.5 psi was maintained. The ionization potential of mass spectrometer (MS) was 70 eV with scan range 20.1 to 350 m/z. The identification of volatiles was accomplished using the Wiley Library (Hewlett-Packard Co.). The area of each peak was integrated using chemstation TM software (Hewlett-Packard Co.) and peak area (total ion counts × 10^4^) was calculated as an indicator of volatiles generated from meat samples.

### Protein oxidation (Total carbonyl) of meat patties

The protein oxidation of patties at various storage intervals was measured by adopting the protocol of [10] with minor modifications. Purposely, 1 g sample was homogenized with Brinkman Polytron (Brinkman Instrument Inc., Westbury, NY, USA) in 10 mL of pyrophosphate buffer (2.0 mM Na4P2O7, 10 mM Trizma-maleate), 100 mM KCL, 2.0 mM MgCl2, and 2.0 mM ethylene glycol tetraacetic acid, pH 7.4). Two equal amounts of meat homogenate (2 mL) were taken from sample, precipitated with 2 mL of 20% trichloroacetic acid and centrifuged at 12,000 × g for 5 min at room temperature. After centrifugation, the pellet from 1 sample was treated with 2 mL of 10 mM 2,4-dinitrophenylhydrazine dissolved in 2 M HCL and the pellet from other incubated with 2 M HCL designated as blank. The samples were vortex-mixed for 10 s after every 3 min during 30 min incubation in the dark. Afterwards, the protein was precipitated with 2 mL of 20% trichloroacetic acid and centrifuged at 12,000 × g for 5 min. The 2,4-dinitrophenylhydrazine was removed by washing 3 times with 4 mL of 10 mM HCL in 1:1 (vol/vol) ethanol:ethyl acetate, followed by centrifuging at 12,000 × g for 5 min. The pellets were finally solubilized in 2 mL of 6.0 mM guanidine hydrochloride dissolved in 20 mM potassium dihydrogen phosphate (pH = 2.3). The samples were kept at 5 °C overnight and next day, they were centrifuged to remove insoluble materials. The absorbance of supernatant was recorded at 370 nm however absorbance for blank samples were subtracted from their corresponding values. Protein concentration was measured using Protein Assay Kit (Bio-Rad Lab, Hercules, CA, USA) following Microplate assay at 280 nm absorbance (BioTek-Gen5 Microplate data collection & analysis software/BioTek Instruments, Inc., Model S4MLFPTA., Winooski, VT, USA). The carbonyl content was calculated as nmol/mg protein using absorption coefficient of 22,000/M/cm.

### Statistical analysis

Resultant data were analyzed by considered the complete randomized design using statistical package (Statistic 8.1). Moreover, Analysis of variance (ANOVA) was performed to measure the level of significance by following the guidelines of [11]. The Tukey’s multiple range test was involved to estimate the level of significant among treatments and storage days.

## Results and discussion

### Lipid oxidation of cooked meat patties

**Fig. 1:**
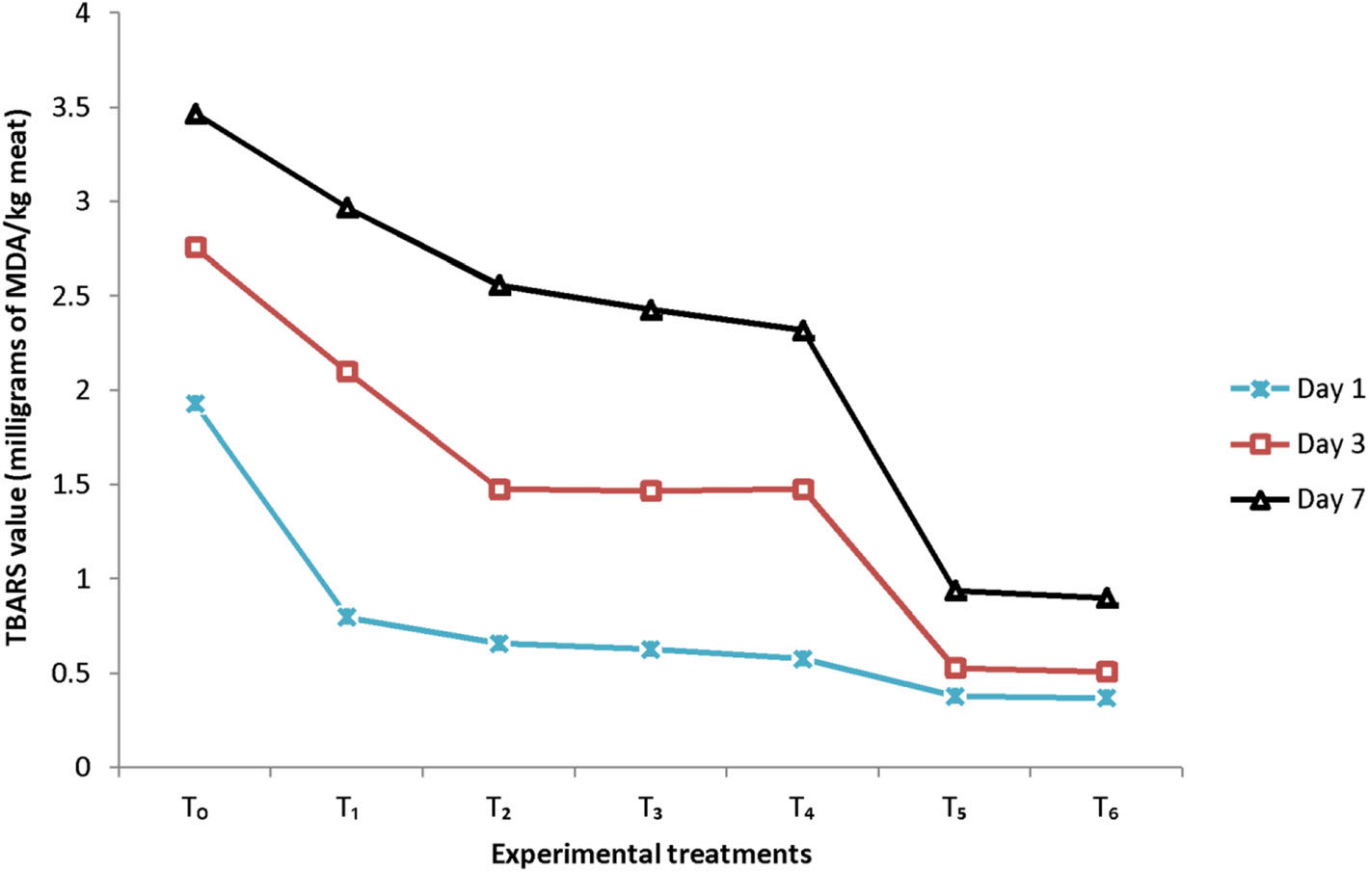
TBARS of cooked breast meat patties stored at refrigeration conditions

The results (Fig. 1) regarding TBARS of quercetin dihdrate and α-tocopherol treated patties delineated significant variations among treatments and storage. At storage initiation, TBARS in various groups T_0_, T_1_, T_2_, T_3_, T_4_, T_5_ and T_6_ were 1.93 ± 0.02, 0.80 ± 0.01, 0.66 ± 0.06, 0.63 ± 0.05, 0.58 ± 0.02, 0.38 ± 0.03 and 0.37 ± 0.01 that subsequently increased to 3.47 ± 0.14 (T_0_), 2.97 ± 0.09 (T_1_), 2.56 ± 0.01 (T_2_), 2.43 ± 0.04 (T_3_), 2.32 ± 0.03 (T_4_), 0.94 ± 0.34 (T_5_), 0.90 ± 0.05 (T_6_) MDA/kg meat correspondingly at storage termination TBARS demonstrated a substantial increase ranged from 0.7641, at initiation to 1.4754 and 2.2283 at 3rd and 7th day of storage, respectively.

During storage in meat based products, deterioration occurs through rancidity resulting from oxidation which takes place at the double bond sites in the triacyleglycerol molecules. It is evident from earlier studies that the oxidation process causes great economic loses to the food industry as well as consumers. Several studies showed that antioxidants especially quercetin dihydrates has ability to enhance the oxidative stability of cooked meat products [12]. The results for storability of nuggets are consistent with [13] noted the effect of antioxidants on TBARS of patties made from antioxidants enriched broiler meat. Likewise, [14] elucidated that antioxidant mixture containing quercetin and rutin at the rate of 0.05 g/ 100 g meat have significant effect on inhibiting the MDA formation during shelf stability study of sausages. Similarly, [15] reported that chicken meat nuggets treated with antioxidants significantly decreased the MDA production however, with storage TBARS increased significantly. The oxidative modification of lipids has long been regarded as a deleterious process responsible for significant changes in the chemical properties of the molecules, loss of function and generation of cytotoxic and genotoxic compounds especially oxidized lipids-derived aldehydes and peroxides. Such lipid peroxidation products have much more stable state and therefore can easily diffuse from their site of generation to remote locations for damage to biological tissues [16, 17]. The repeated consumption of such oxidized fats and fat containing products in the diet poses a chronic threat to human health by selective alterations in cell signaling, protein, DNA damage and dysfunction of organs such as liver, kidney, lung and the gut [18]. However, the oxidative stability of lipid fraction in complex food system is dependent on the composition, concentrations of reaction substrates, prooxidants and antioxidants. Therefore, decreasing the formation of lipid peroxidation products or scavenging them chemically could be beneficial in limiting the deleterious effects of reactive oxygen species in various pathological conditions. This could be potentially achieved by enhancing the endogenous oxidation control systems of foods through supplementation of antioxidants [19, 20].

### Color analysis

**Fig. 2:**
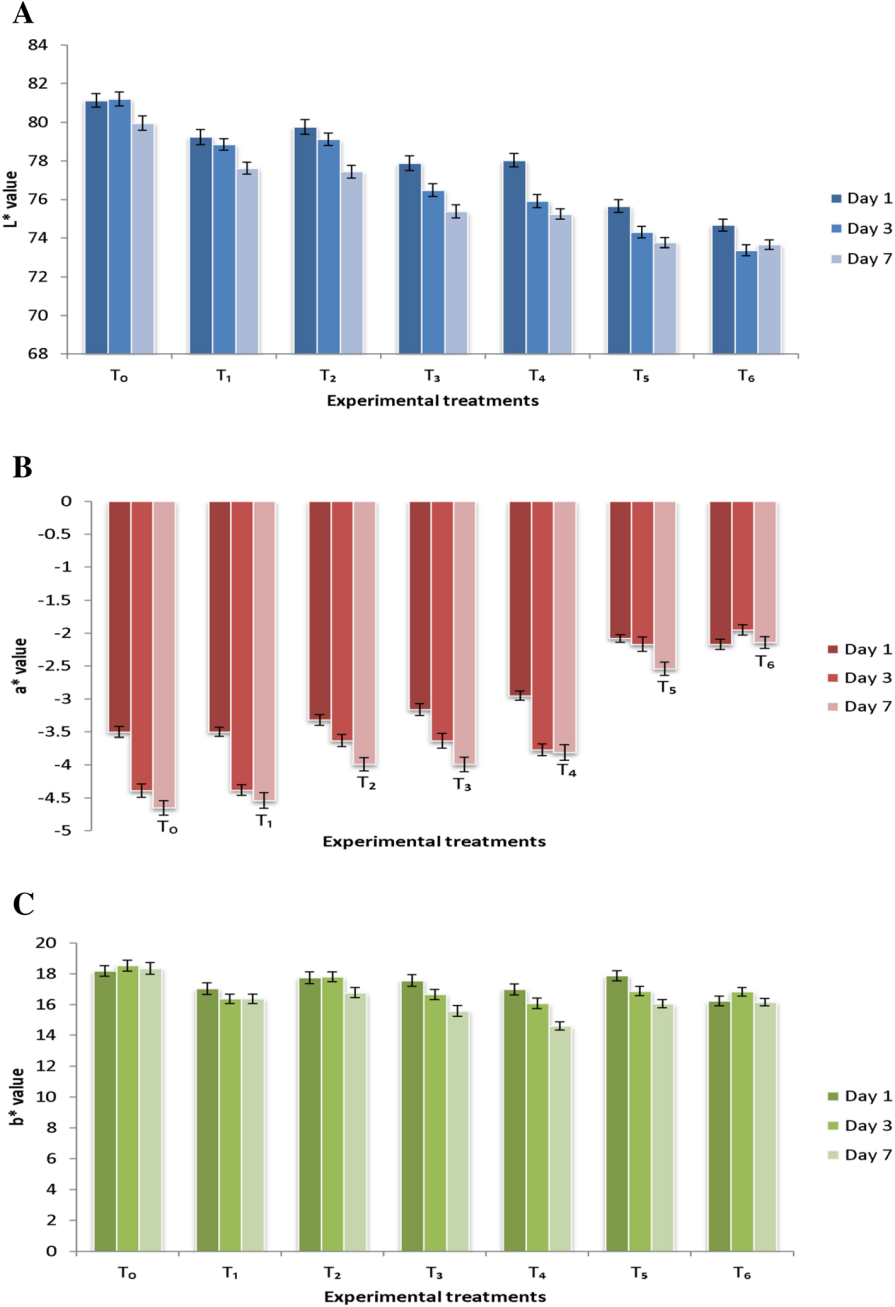
Color values of cooked patties stored at 4 °C **a** *Lightness* (L*), **b** *Redness* (a*) and **c** *Yellowness* (b*)

The Fig. 2 depicted results for color attributes such as lightness (L* − values), redness (a*) and yellowness (b*) that were affected by the treatments application. Among treatments, maximum L* value noticed in T_0_ (control) 80.773 whereas, the minimum 73.903 in T_6_. Likewise, a* value also varied among treatments and with progress storage. Similarly, b* color values at initiation varied from 16.23 ± 1.28 to 18.18 ± 0.23. Current study findings are in agreement with [21], they reported a decrease in redness (a*) for patties with storage due to metmyoglobin accumulation. One of the researchers groups, [22] delineated that addition of vitamin E @200 and 300 ppm improve the visual color in meat product. Likewise, [23] reported that chicken meat balls containing pomegranate rind powder extract as source of antioxidants significantly (*p* < 0.05) higher color nonetheless, this value decreased with storage. Similarly, [24] recorded lower discoloration (*p* < 0.05) in longissimus lumborum muscles for lambs meat fed on quercetin alone or in combination with flaxseed.

### Protein oxidation

The functional properties of proteins such as solubility, gelation and emulsification potential in different food products depend on their amino acid composition and structural arrangement. Oxidative deterioration of amino acids mainly lysine, proline, arginine and histidine can generate the carbonyl compounds that can affect the functionality of meat proteins especially in cooked meat products and antioxidants addition can decrease the rate of protein oxidation [25]. The results indicated that quercetin dihydrate and α-tocopherol significantly (*p* < 0.05) reduced the formation of the total carbonyl. The concentration of proteins carbonyls increased (*p* ≤ 0.05) treated groups. The results (Table 1) indicated that addition of quercetin dihydrate at the rate of 100 ppm along with α-tocopherol at the concentration of 100 & 200 ppm showed the strongest potential in reducing total carbonyls and means reported lowest total carbonyls in T_6_ (0.7922) trailed by T_5_ and T_4_ (0.9256 & 1.3100) whereas, highest in T_0_ (1.7244). The formation of total carbonyls was consistent with TBARS in patties with storage progression and results are in harmony with [26] who reported that antioxidants addition decreased the formation of total carbonyls. Similarly, [27] indicated that mincing and high temperatures cooking can increase oxidation of protein in patties.

**Table 1:** Protein oxidation of cooked breast meat patties stored at refrigeration temperature

Storage	T_0_	T_1_	T_2_	T_3_	T_4_	T_5_	T_6_
Day 1	0.71 ± 0.015^e^	0.70 ± 0.021^e^	0.69 ± 0.020^e^	0.66 ± 0.015^f,h^	0.65 ± 0.015^f,h^	0.63 ± 0.015^h^	0.59 ± 0.025^i^
Day 3	1.55 ± 0.026^d^	1.42 ± 0.021^d,e^	1.37 ± 0.015^e^	1.33 ± 0.036^e,f^	1.25 ± 0.015^f^	0.88 ± 0.031^g^	0.77 ± 0.015^g,e^
Day 7	2.92 ± 0.060^a^	2.83 ± 0.067^a,b^	2.77 ± 0.038^b^	2.16 ± 0.050^c^	2.03 ± 0.026^c,d^	1.26 ± 0.050^f^	1.02 ± 0.031^f,g^

^a–i^Means within a row or column with different superscript are statistically significant to each other (*p* > 0.05)

### Volatiles compounds in chicken meat patties

The generation of off flavor is critical problem that can affect storage stability of cooked meat products [28]. The oxidation of polyunsaturated fatty acids results in the generation of volatile compounds which many have unpleasant odors and are responsible for the off-flavors in the food products [29]. Polyunsaturated fatty acids oxidation generates volatile compounds that impart undesirable aromas and lead towards compromising the nutritional quality of the meat and meat products with limited shelf life [30]. Oxidation is considered as one of the most common causes of flavor quality deterioration for meat based products during storage which accounts the great economic loses to the food industry and allied consumers. Adding α-tocopherol and quercetin dehydrate significantly diminish the generation of off-odor volatiles in poultry meat (Tables 2, 3 and 4). Major volatiles identified in cooked patties were hydrocarbons, aldehydes, ketones, alcohols and sulfur compounds generated due to degradation of fatty acids and amino acids [31].

**Table 2:** Volatile profile of cooked breast meat patties on 1st day of storage

Compound	T_0_	T_1_	T_2_	T_3_	T_4_	T_5_	T_6_
Pentane	3013 ± 188^c^	33,820 ± 1549^b,c^	49,361 ± 1669^a,b,c^	85,936 ± 1099^a,b^	30,246 ± 1667^b,c^	71,611 ± 1969^a,b^	104,062 ± 1824^a^
Heptane	3808 ± 129^a^	2405 ± 198^a,b^	573 ± 49^c^	866 ± 56^c^	771 ± 65^c^	349 ± 29^c^	986 ± 83^b,c^
1-octene	352 ± 31^c^	2239 ± 109^a^	927 ± 52b^c^	441 ± 37^c^	1029 ± 81^b,c^	1012 ± 92^b,c^	1810 ± 152^a,b^
Octane	32,775 ± 976^a^	27,636 ± 1058^a^	11,151 ± 406^b^	6439 ± 413^b^	8238 ± 377^b^	6745 ± 164^b^	7861 ± 202^b^
2-octene	191 ± 11^b^	2044 ± 103^a^	1020 ± 85^a,b^	1233 ± 64^a,b^	1222 ± 70^a,b^	1823 ± 71^a^	2533 ± 219^a^
1-heptene	0 ± 0	0 ± 0	0 ± 0	0 ± 0	0 ± 0	0 ± 0	0 ± 0
2-propanone	12,309 ± 289^a^	12,328 ± 266^a^	8047 ± 224^a^	7990 ± 207^a^	7081 ± 237^a^	6598 ± 201^a^	6408 ± 163^a^
2-Butanone	0 ± 0^a^	0 ± 0^a^	1562 ± 121^a^	1485 ± 88^a^	0 ± 0^a^	0 ± 0^a^	0 ± 0^a^
Propanal	0 ± 0	0 ± 0	0 ± 0	0 ± 0	0 ± 0	0 ± 0	0 ± 0
Butanal	3618 ± 98^a^	3257 ± 199^a^	1475 ± 121^b^	703 ± 52^b^	3399 ± 152^a^	3253 ± 179^a^	3462 ± 91^a^
Pentanal	11,927 ± 380^a^	3391 ± 104^b^	2431 ± 96^b^	1874 ± 45^b^	1626 ± 69^b^	939 ± 63^b^	1222 ± 45^b^
Hexanal	54,768 ± 431^a^	11,568 ± 283^b^	8902 ± 383^b^	7367 ± 318^b^	3701 ± 111^b^	3759 ± 34^b^	2488 ± 103^b^
Heptanal	1737 ± 66^a^	0 ± 0^b^	0 ± 0^b^	0 ± 0^b^	0 ± 0^b^	0 ± 0^b^	0 ± 0^b^
Octanal	97 ± 7^a^	0 ± 0^a^	0 ± 0^a^	0 ± 0^a^	0 ± 0^a^	0 ± 0^a^	0 ± 0^a^
Nonanal	806 ± 30^a^	0 ± 0^b^	0 ± 0^b^	0 ± 0^b^	0 ± 0^b^	0 ± 0^b^	0 ± 0^b^
Alcohol							
Cyclopentanol	0 ± 0	0 ± 0	0 ± 0	0 ± 0	0 ± 0	0 ± 0	0 ± 0
Dimethyldisulfide	4037 ± 133^a,b^	5907 ± 182^a^	2596 ± 216^b^	2081 ± 239^b^	3410 ± 217^a,b^	2215 ± 105^b^	3918 ± 175^a,b^
Benzoic acid	364 ± 26^a^	513 ± 44^a^	0 ± 0^b^	0 ± 0^b^	0 ± 0^b^	0 ± 0^b^	0 ± 0^b^
1,3-octadiene	0 ± 0	0 ± 0	0 ± 0	0 ± 0	0 ± 0	0 ± 0	0 ± 0

^a–c^Means with in a row with different superscript are statistically significant to each other (*p* > 0.05)

**Table 3:** Volatile profile of cooked patties on 3rd day of storage

Compound	T_0_	T_1_	T_2_	T_3_	T_4_	T_5_	T_6_
Pentane	12,877 ± 79^d^	28,958 ± 128^c,d^	83,941 ± 145^a^	68,506 ± 149^a,b,c^	22,902 ± 132^c,d^	76,007 ± 1710^a,b^	40,153 ± 106^a,b,c,d^
Heptane	1037 ± 53^c^	3244 ± 81^a^	2302 ± 26^a,b,c^	2865 ± 36^a,b^	1875 ± 26^a,b,c^	998 ± 34^c^	1584 ± 31^b,c^
1-octene	0 ± 0^b^	1337 ± 61^a^	1276 ± 56^a^	1237 ± 84^a^	1505 ± 75^a^	1198 ± 46^a^	1459 ± 35^a^
Octane	10,753 ± 149^b,c,d^	16,792 ± 183^a^	13,030 ± 107^a,b,c,d^	16,181 ± 123^a,b^	14,604 ± 104^a,b,c^	7444 ± 407^d^	10,071 ± 98^c,d^
2-octene	729 ± 36^d^	3690 ± 116^a,b^	1586 ± 66^c,d^	1970 ± 83^c,d^	1835 ± 78^c,d^	2412 ± 86^b,c^	4666 ± 116^a^
1-heptene	109 ± 9^a^	94 ± 7^a^	0 ± 0^a^	0 ± 0^a^	0 ± 0^a^	0 ± 0^a^	0 ± 0^a^
2-propanone	18,196 ± 166^a,b,c^	16,417 ± 124^c^	24,778 ± 253^a^	20,207 ± 136^a,b,c^	23,353 ± 139^a,b^	19,291 ± 132^a,b,c^	17,417 ± 109^b,c^
2-Butanone	5650 ± 62^a,b^	7274 ± 88^a^	6263 ± 85^a,b^	7534 ± 97^a^	9198 ± 170^a^	6257 ± 107^a,b^	0 ± 0^b^
Propanal	6830 ± 189^a^	3726 ± 104^a,b,c^	5078 ± 111^a,b^	5971 ± 115^a^	4244 ± 121^a,b^	1541 ± 91^c^	1092 ± 83^b,c^
Butanal	8148 ± 274^a^	6656 ± 60^b^	7201 ± 145^a,b^	8099 ± 201^a^	7220 ± 174^a,b^	4223 ± 140^c^	5229 ± 110^c^
Pentanal	61,228 ± 1134^a^	39,879 ± 936^b^	33,392 ± 531^b^	38,633 ± 596^b^	31,788 ± 477^b^	4467 ± 92^c^	7550 ± 391^c^
Hexanal	263,841 ± 1239^a^	203,916 ± 2406^b^	184,930 ± 1765^b^	181,390 ± 1446^b^	161,374 ± 1088^b^	18,259 ± 427^c^	9136 ± 307^c^
Heptanal	7313 ± 53^a^	3892 ± 79^b^	3909 ± 60^b^	3379 ± 89^b^	2896 ± 75^b^	0 ± 0^c^	0 ± 0^c^
Octanal	933 ± 52^a^	345 ± 29^b^	461 ± 37^b^	371 ± 24^b,c^	285 ± 17^b,c^	0 ± 0^c^	0 ± 0^c^
Nonanal	1092 ± 65^a^	738 ± 37^a,b^	731 ± 43^a,b^	664 ± 46^b^	633 ± 42^b^	0 ± 0^c^	0 ± 0^c^
Cyclopentanol	0 ± 0	0 ± 0	0 ± 0	0 ± 0	0 ± 0	0 ± 0	0 ± 0
Dimethyldisulfide	4968 ± 126^b,c^	2633 ± 143^c,d^	7229 ± 194^a,b^	8168 ± 190^a^	6123 ± 154^a,b^	2524 ± 148^d^	588 ± 42^d^
Benzoic acid	743 ± 34^a^	660 ± 24^a,b^	352 ± 21^b,c^	271 ± 12^c^	0 ± 0^c^	0 ± 0^c^	0 ± 0^c^
1,3-octadiene	0 ± 0^b^	462 ± 23^a^	363 ± 29^a^	418 ± 12^a^	349 ± 28^a^	0 ± 0^b^	0 ± 0^b^

^a–d^Means with in a row with different superscript are statistically significant to each other (*p* > 0.05)

**Table 4:** Volatile profile of cooked patties on 7th day of storage

Compound	T_0_	T_1_	T_2_	T_3_	T_4_	T_5_	T_6_
Pentane	14,677 ± 177^b^	27,436 ± 128^a,b^	48,133 ± 182^a,b^	31,390 ± 197^a,b^	23,013 ± 101^a,b^	71,928 ± 317^a,b^	78,607 ± 319^a^
Heptane	5114 ± 168^a^	3916 ± 114^a,b^	3531 ± 124^b,c^	1985 ± 140^d^	2604 ± 109^c,d^	2002 ± 72^d^	1557 ± 63^d^
1-octene	0 ± 0^d^	1200 ± 31^b,c^	822 ± 47^b,c,d^	355 ± 28^c,d^	593 ± 26^c,d^	1789 ± 38^a,b^	2337 ± 41^a^
Octane	13,920 ± 181^a,b^	16,972 ± 174^a^	12,739 ± 176^a,b,c^	9029 ± 180^c,d^	11,830 ± 153^b,c,d^	11,574 ± 168^b,c,d^	8083 ± 107^d^
2-octene	1075 ± 44^c^	3327 ± 48^a,b^	2111 ± 26^b,c^	1383 ± 24^c^	1841 ± 37^b,c^	3368 ± 57^a,b^	4505 ± 35^a^
1-heptene	291 ± 19^a,b^	547 ± 22^a^	210 ± 15^a,b^	129 ± 11^b^	120 ± 9^b^	0 ± 0^b^	0 ± 0^b^
2-propanone	15,113 ± 104^c^	17,980 ± 114^c^	21,044 ± 195^b,c^	20,689 ± 107^b,c^	27,303 ± 163^a,b^	32,990 ± 221^a^	33,102 ± 228^a^
2-Butanone	5661 ± 96^b^	7826 ± 82^b^	9716 ± 87^a,b^	9680 ± 85^a,b^	11,751 ± 101^a,b^	14,666 ± 107^a^	10,120 ± 84^a,b^
Propanal	8421 ± 142^a^	6060 ± 169^a^	7241 ± 189^a^	7241 ± 133^a^	9038 ± 154^a^	6115 ± 157^a^	2948 ± 91^a^
Butanal	10,892 ± 131^a^	8833 ± 126^a,b,c^	9592 ± 145^a,b^	8321 ± 229^b,c,d^	9387 ± 152^a,b^	7037 ± 121^c,d^	6153 ± 134^d^
Pentanal	96,422 ± 304^a^	65,251 ± 384^b^	69,319 ± 335^b^	56,315 ± 3432^b^	59,438 ± 314^b^	21,064 ± 247^c^	13,840 ± 186^c^
Hexanal	359,826 ± 1285^a^	279,277 ± 1048^b,c^	286,879 ± 1048^b^	239,163 ± 1104^c^	244,203 ± 999^c^	112,550 ± 897^d^	9569 ± 607^e^
Heptanal	13,046 ± 118^a^	7073 ± 81^b^	8339 ± 73^b^	5764 ± 65^b^	6409 ± 67^b^	1744 ± 46^c^	330 ± 23^c^
Octanal	2455 ± 46^a^	856 ± 34b^c^	1726 ± 32^a,b^	633 ± 26^c^	949 ± 37^b,c^	0 ± 0^c^	0 ± 0^c^
Nonanal	1974 ± 21^a^	1530 ± 27^a,b^	1406 ± 23^b,c^	983 ± 16^c^	1082 ± 39^b,c^	313 ± 18^d^	0 ± 0^d^
Cyclopentanol	1682 ± 33^a^	788 ± 23^b^	658 ± 26^b,c^	247 ± 17^c,d^	161 ± 9^d^	0 ± 0^d^	0 ± 0^d^
Dimethyldisulfide	4159 ± 146^a^	3843 ± 132^a^	5492 ± 154^a^	3427 ± 147^a^	4454 ± 113^a^	3263 ± 101^a^	2916 ± 75^a^
Benzoic acid	889 ± 24^a^	916 ± 28^a^	480 ± 14^a,b^	263 ± 15^b,c^	678 ± 31^a,b^	0 ± 0^c^	0 ± 0^c^
1,3-octadiene	193 ± 11^b^	421 ± 21^a,b^	847 ± 26^a^	88 ± 7^b^	813 ± 19^a^	352 ± 22^b^	412 ± 14^a,b^

^a–e^Means with in a row with different superscript are statistically significant to each other (*p* > 0.05)

Hydrocarbons are among the major group of volatiles that can affect flavor of cooked products under storage. Major hydrocarbons reported in treated patties were pentane, heptane, 1-octene, octane, 2-octene and 1-heptene. The generation of hydrocarbon was increased with progression of storage however, hydrocarbons increased linearly with quercetin dihydrate and α-tocopherol in a dose dependent manner. On 1st day, Pentane, heptane, 1-octene, octane and 2-octene were ranged between 3013 ± 188 to 104,062 ± 1824, 349 ± 29 to 3808 ± 129, 352 ± 31 to 2239 ± 109, 6439 ± 213 to 32,775 ± 976, 191 ± 11 to 2533 ± 109, correspondingly. Heptane and octane were decreased with increasing level of antioxidants. However, storage progression, their amount was increased. 1-Heptane was not detected on 1st day, however, 2-octene did not show clear trend but overall lower values were reported with increasing antioxidants concentration in meat samples. Likewise, on 7th day highest pentane was recorded in T_6_ (78,607 ± 319) followed by T_5_ (71,928 ± 317) and lowest in T_0_ (14,677 ± 177), respectively.

The results showed aldehydes in patties were propanal, butanal, pentanal, hexanal, heptanal, octanal and nonanal and their concentration significantly (*p* < 0.05) decreased with increasing level of quercetin dihydrate and α-tocopherol. At initiation of storage, maximum hexanal were reported in T_0_ (control) and lowest in T_5_ and T_6_ containing 100 ppm quercetin dihydrate such as 54,768 ± 1431, 3701 ± 134, 2488 ± 103 that were significantly increased to 359,826 ± 1285, 112,550 ± 16,709, 90,569 ± 607, respectively at storage termination. Similarly other volatiles like propanol, heptanal, octanal and nonanal were not detected in antioxidant added treatments and aldehydes showed a positive linear correlation with storage and treatments. Likewise, on 1st day lowest butanal and pentanal were found in T_6_ followed by T_5_, and T_4_ and highest in T_0_ as 3462 ± 91 & 1222 ± 45, 3253 ± 179 & 939 ± 63, 3399 ± 152 & 1626 ± 69, 3618 ± 98 & 11,927 ± 380, correspondingly that were increased to 6153 ± 134 & 13,840 ± 186, 7037 ± 121 & 21,064 ± 247, 9387 ± 152 &59,438 ± 314, 10,892 ± 131 & 96,422 ± 304, respectively at the termination of the storage.

Among ketone group, only 2-propanone and 2-butanone were recorded in patties samples. The results further indicated that on 1st day, lowest 2-propanone was found in T_6_ (6408 ± 163) followed by T_5_ and T_4_ that were 6598 ± 201, 7081 ± 237 and highest in T_0_ as 12,309 ± 289 that were increased to 33,102 ± 228, 32,990 ± 221, 27,303 ± 163 and 15,113 ± 104 at termination of storage. 2-Butanone results showed that on day 1st, they were detected in T_2_ and T_3_ only while at the completion of storage, lowest were found in T_0_ and highest in T_5_ as 5661 ± 96, 14,666 ± 107, correspondingly. Similarly, others volatiles reported in patties were dimethyldisulfide, benzoic acid and 1,3-octadiene. Dimethyl sulfide content decreased with storage however, benzoic acid and 1,3-octadiene were not consistent among treatments and with storage. Besides, different does of quercetin dehydrate and alpha tocopherol in meat patties, storage is also a prominent factor that influenced the production of off flavor volatile compounds and its production especially aldehyde including hexanal, pentnal, butanal, heptanal etc. increased with storage production. The incorporation of antioxidants significantly reduces the rate of volatiles generation and current study results are supported by [32] who reported positive relation between aldehyde compounds and TBARS in chicken meat. Similarly, [33] also narrated that aldehyde presence in cooked meat is mainly attributed to lipid degradation that is enhanced with cooking and storage. Hexanal, an important aldehyde that can generate grassy aroma note [34] is among the most abundant aldehyde fund in meat based products. This hexanal is mostly generated from fatty acids degradation as poultry meat is rich source of polyunsaturated fatty acids [35]. Similarly, [36] noticed sulfur volatiles escaped after 5 day storage due to their high volatility.

## Conclusion

Current study findings revealed that addition of quercetin dihdrate alone as well as in combination with α-tocopherol improved oxidative stability, total carbonyls as well as volatile off flavor compounds in treated chicken patties. Quercetin dihdrate incorporation at a level of 100 mg/kg meat with α-tocopherol at the rate of 100 and 200 mg/kg meat delayed lipid and protein degradation by inhibiting oxidation of cooked meat products. Additionally, quercetin dihydrate addition also decreased the aldehydes volatiles particular hexanal and pentanal that are considered as a major index to judge the storability of cooked meat products. Furthermore, preliminary studies showed that using quercetin along gives better oxidative and volatiles generation results than that of α-tocopherol however, quercetin alone imparts yellowness thereby using it in combination with α-tocopherol is a better choice to enhance the oxidative stability and quality of processed poultry meat products. The current exploration concluded quercetin dihdrate along with α-tocopherol is a pragmatic choice to enhance the oxidative stability by inhibiting the production of malonaldehydes and total carbonyls as well as volatile flavor compounds in cooked chicken meat products.
